# Suitability of MRF Recovered Post-Consumer Polypropylene Applications in Extrusion Blow Molded Bottle Food Packaging

**DOI:** 10.3390/polym15163471

**Published:** 2023-08-19

**Authors:** Ma. Cristine Concepcion D. Ignacio, Khairun N. Tumu, Mita Munshi, Keith L. Vorst, Greg W. Curtzwiler

**Affiliations:** 1 Polymer and Food Protection Consortium, Iowa State University, Ames, IA 50011, USA; mignacio@iastate.edu (M.C.C.D.I.); kntumu@iastate.edu (K.N.T.); mmunshi@iastate.edu (M.M.); kvorst@iastate.edu (K.L.V.); 2 Department of Agricultural and Biosystems Engineering, Iowa State University, Ames, IA 50011, USA; 3 Department of Food Science and Human Nutrition, Iowa State University, Ames, IA 50011, USA

**Keywords:** extrusion blow molding, municipal recovery facility, polypropylene, polymer recycling, post-consumer recycling, polymer processing

## Abstract

Polypropylene (PP) is one of the most abundant plastics used due to its low price, moldability, temperature and chemical resistance, and outstanding mechanical properties. Consequently, waste from plastic materials is anticipated to rapidly increase with continually increasing demand. When addressing the global problem of solid waste generation, post-consumer recycled materials are encouraged for use in new consumer and industrial products. As a result, the demand is projected to grow in the next several years. In this study, material recovery facility (MRF)-recovered post-consumer PP was utilized to determine its suitability for extrusion blow molded bottle food packaging. PP was sorted and removed from mixed-polymer MRF-recovered bales, ground, trommel-washed, then washed following the Association of Plastics Recyclers’ protocols. The washed PCR-PP flake was pelletized then manually blended with virgin PP resin at 25%, 50%, 75, and 100% PCR-PP concentrations and fed into the extrusion blow molding (EBM) machine. The EBM bottles were then tested for physical performance and regulatory compliance (limits of TPCH: 100 μg/g). The results showed an increased crystallization temperature but no practical difference in crystallinity as a function of PCR-PP concentrations. Barrier properties (oxygen and water vapor) remained relatively constant except for 100% MRF-recovered PCR-PP, which was higher for both gas types. Stiffness significantly improved in bottles with PCR-PP (*p*-value < 0.05). In addition, a wider range of N/IAS was detected in PCR-PP due to plastic additives, food additives, and degradation byproducts. Lastly, targeted phthalates did not exceed the limits of TPCH, and trace levels of BPA were detected in the MRF PCR-PP. Furthermore, the study’s results provide critical information on the use of MRF recovered in food packaging applications without compromising performance integrity.

## 1. Introduction

Increasing plastic production continues to amplify the negative impact of global solid waste management on the environment and human health. Plastic production reached 390.7 million tons in 2021, consisting of 90% fossil-based plastics, 8.3% post-consumer recycled plastics, and 1.5% bio-based plastics [1]. Among the four major plastics (polyethylene (PE), polypropylene (PP), polystyrene (PS), and polyethylene terephthalate (PET)) used in packaging, PP is considered to be one of the most abundant plastics used worldwide [2,3]. In 2015, the global PP production was about 68 million metric tons, generating about 55 million metric tons of plastic waste [3]. Polypropylene is used in a wide range of applications due to its low price, moldability, temperature and chemical resistance, and outstanding mechanical properties [2,4]. Thus, PP production is anticipated to grow rapidly over the next decade to USD 108.57 billion at a CAGR of 5.2% from 2021 to 2028 [5]. Consequently, global solid-waste generation will continue to increase, along with its environmental impacts, which affect soil and water resources because of plastic landfill disposal and accumulation.

The recycling process is one notable solution to minimizing plastic waste and significantly contributes to promoting circular economy approaches, which include designing products for reuse, finding innovative techniques for recycling plastics, and incorporating PCR products [1]. Worldwide efforts and programs have been initiated and implemented to encourage the recycling and upcycling of plastic products. In the United States (U.S.), the Environmental Protection Agency (EPA) coordinated the development of the National Recycling Strategy in 2021. The goal is to increase the U.S. recycling rate to 50% by 2030 by improving markets for recycling commodities, improving infrastructure, reducing contamination, enhancing policies to support circularity, and standardizing measurement and data collection [6]. On the other hand, the findings of the “Circular Economy for Plastics 2020 EU27 + 3” (Norway, Switzerland, and the United Kingdom) study showed that the recycling rate increased to nearly 35%, while an increase of 1.3 percentage points was obtained for plastic parts and products with post-consumer recycled (PCR) content [7].

Furthermore, the benefits of and perspectives on plastic recycling have been established in published studies. Plastics recycling has challenges not limited to the presence of contaminants in direct food-contact applications [8], inadequate recycling infrastructure and sorting systems for certain types of plastics, such as polypropylene [2,9], high costs and economic viability, and limited demand for recycled plastics. In the United States, only 9% of the majority of plastics wastes (PE, PP, PS, and PET) was mechanically recycled in 2018 [3], and this fell to a rate of 5–6% in 2020 [10]. Despite all developments worldwide, more effort must be made to improve collection, sorting, and recycling technologies to sufficiently meet the targets [6,7]. Due to PP’s wide range of applications, its waste can have very different properties and contaminants [2]; thus, the recycling process can be challenging. In addition, PP and PE are immiscible plastics that are difficult to separate with current recycling practices [9], which raised the question of how to efficiently recover high-value post-consumer plastics with high purity from single-stream collection. To address this matter and improve the recycling rate and systems, the European Union (EU) has enforced Extended Producer Responsibility (EPR) laws on packaging products since 1994, a recognized efficient waste-management policy that has become more popular in the U.S. Research studies have reported different techniques and processes to recover PP wastes [2], multilayer packaging films [11], plastic waste from material recovery facilities [12], and mixed wastes [13], aiming to achieve climate change mitigation and a circular economy.

PCR materials are known to play a vital role in sustainable packaging strategies. Studies have predicted a high market potential for recycled PP, with demands for increases in post-consumer polypropylene (PCR-PP) and global brands showing more interest in the inclusion of PCR-PP in their products [14,15]. With the enforcement of new laws and regulations, awareness of sustainable plastic waste management and reductions in environmental impacts through plastic recycling are increasing. One such initiative is the Polypropylene Recycling Coalition, which, in two years, granted 24 MRFs to acquire upgrades in sorting technology and produce high-quality recycled PP for reuse in packaging [16]. However, one of the major issues in using recycled materials is their suitability for direct food-contact applications and impact on the packaging integrity and performance. Single-stream and material recovery facility (MRF)-recovered plastics with food- and nonfood-grade materials can introduce unapproved additives and contamination to food-grade materials. Thus, pre-sorting becomes necessary to reduce potential human exposure risk to PCR in food packaging or to obtain single-sourced materials from known food-grade applications. The Toxics in Packaging Clearinghouse Model legislation prohibits the intentional use of four metals (cadmium, lead mercury, and hexavalent chromium) in any finished package, with a combined limit of 100 ppm [17]. In 2021, the legislation added the class of perfluoroalkyl and poly-fluoroalkyl substances (PFAS) and ortho-phthalate as regulated chemicals in the packaging [18]. Thus, an effective risk assessment is important to identify undesired substances and their sources in PCR plastics to limit contamination and ensure compliance. In addition, the main requirements for a material to be used in packaging often include barriers to oxygen and water vapor and proper mechanical properties to ensure package performance [19]. Lastly, an evaluation of the mechanical, thermal, and barrier performance of plastic packaging, incorporated with PCR plastics, must be accurately measured and documented.

This research aimed to determine the compliance and physical performance of extrusion blow-molded MRF-recovered PCR-PP bottles. The results of this research provide baseline information on the viability of using MRF-recovered PCR-PP while maintaining properties and ensuring compliance for direct food-contact applications.

## 2. Materials and Methods

### 2.1. Materials and Bottle Manufacturing Process

Post-consumer polypropylene was collected from a sorted #3–7 (resin identification codes) bale that could include polyvinyl chloride (PVC; #3), low-density polyethylene (LDPE; #4), polypropylene (PP; #5), polystyrene (PS; #6), and other (#7), sourced from a material recovery facility (MRF) located in Iowa, USA. Unwashed post-consumer PP flakes were washed following a typical wash procedure in a commercial recycling facility [20]. The PP flakes were washed in a laboratory-sized trommel separator with water to remove contaminants such as glass, woods, paper, and metals using the APR detergent wash solution (0.5% wt. of NaOH and 0.3% wt. of Triton X-100) in a five-gallon stainless-steel tank with four baffles [20]. The washed flake was then rinsed with water and dried.

**Figure 1:**
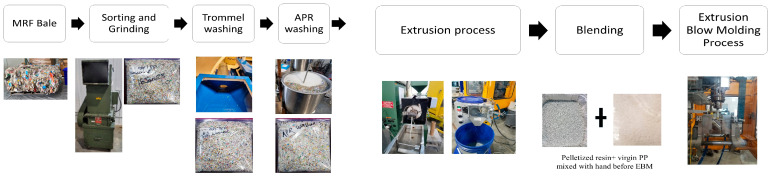
MRF-recovered PCR-PP to bottle manufacturing process.

Figure 1 shows the MRF bale-to-washing-to-bottle manufacturing process conducted in this study. The dried flakes from the washing process were then pelletized using a micro 18GL 18 mm twin-screw-co-rotating extruder (Leistritz, Sommerville, NJ, USA) and lab-scale pelletizer BT 25 (Bay Plastics Machinery, Bay City, MI, USA).

**Figure 2:**
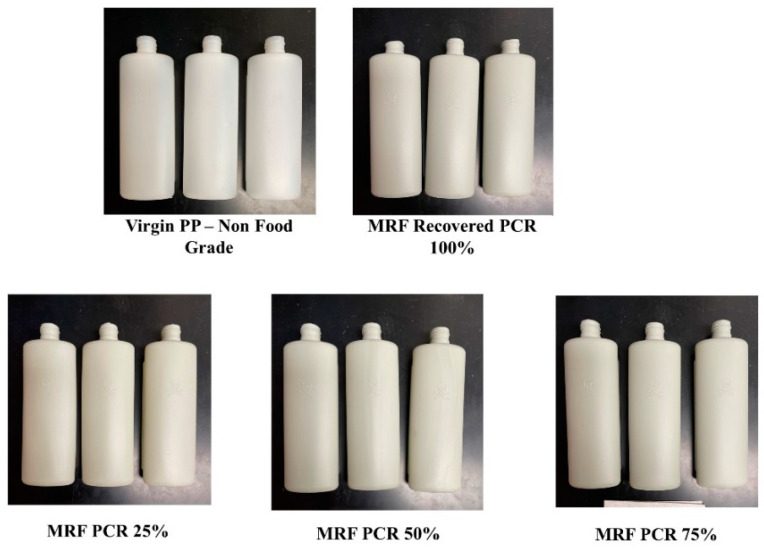
Optical images of the PCR-PP extrusion blow model bottles used in the study.

Virgin PP resin was a non-food-grade polypropylene copolymer manufactured by Huntsman (Huntsman Copolymer PP RX PP18S07A-G Lot#A103HPP3048). The pelletized MRF-recovered PCR-PP pellets were manually mixed with virgin PP resin at different weight percentages (25%, 50%, 75%, and 100%); then, at least 20 bottles were manufactured using an Extrusion Blow Molder Model CS1 (Rocheleau Tool and Die Company, Fitchburg, MA, USA). Figure 2 shows the PCR-PP EBM bottles produced and analyzed in this study. The optimized EBM parameters used for PCR-PP bottles are listed in Table S1 (Supplementary Data).

A complete randomized design (CRD) was used in this study (Table 1). Five treatments correspond to different weight concentrations of MRF-recovered PCR-PP mixed with PP virgin resin. For each treatment, at least three EBM bottles, as repeated measures, were used for physical performance tests (viscosity, molecular structure, thermal, barrier and tensile properties) and suitability analyses (heavy metals and CFR analysis) for direct food-contact applications.

### 2.2. Characterizing MRF-Recovered Post-Consumer PP EBM Bottles

#### 2.2.1. Melt Flow Index

The melt flow rate of MRF-recovered post-consumer PP EBM bottles and virgin PP bottle was obtained in accordance with Procedure A of ASTM D1238–20 [21] in three repeated measures. The test utilized the specified parameters for polypropylene (230 °C, 2.16 kg) using a D4004 Melt Flow Indexer (Dynisco, Morgantown, PA, USA). Approximately 3.0–8.0 g of sample was loaded into the cylinder and the material was pre-heated for 6 min. The cut-off time was different for each of the samples depending on the flow rate. By applying the appropriate factor, the mass was then converted into grams per 10 min [22].

#### 2.2.2. Infrared Spectroscopy

Attenuated total reflectance Fourier-Transform Infrared Spectroscopy (ATR-FTIR) was used to determine changes in chemical bonding and interactions of post-consumer PP bottles and virgin PP bottles in three repeated measures. A Nicolet 6700 infrared spectrometer (Thermo Scientific, Waltham, MA, USA) with a DTGS detector was used with 32 scans per run and a 2 cm^−1^ resolution at ambient temperature (22 °C). All spectra were baseline-corrected with OMINIC ^TM^ 8.3 software (Thermo Fisher, Waltham, MA, USA) [22]. The diamond window of the ATR assembly was cleaned with an isopropanol wipe after each measurement to ensure no cross-contamination [23].

#### 2.2.3. Differential Scanning Calorimetry

A Q2000 differential scanning calorimeter (TA Instruments, New Castle, DE, USA) was used to investigate the thermal transition properties for each PCR-PP and virgin PP bottle blend using a heat/cool/heat cycle between −10 °C and 310 °C, at a rate of 10 °C/min, under an N_2_ atmosphere. Each treatment was tested with three repeated measurements with a sample mass of from 3 to 6 mg in a hermetically sealed aluminum DSC pan, according to ASTM D3418–15 [24]. An empty pan was used as a reference.

#### 2.2.4. Oxygen Induction Time

The oxidation induction time (OIT) was used to evaluate the thermo-oxidative stability of the polymer blends. The OIT tests were carried out according to ASTM D3895 [25] using a Q2000 differential scanning calorimeter (TA Instruments, New Castle, DE, USA). A mass of 3–6 mg was placed in an open standard aluminum DSC pan, while an empty pan was used as reference. The sample was initially heated from 50 °C to 200 °C at a heating rate of 10 °C/min, under a nitrogen flow of 50 mL/min. Once the temperature reached 200 °C, the sample was isothermally conditioned for 5 min; then, the atmosphere switched from nitrogen to air. The sample was held at 200 °C until the sample went through oxidative degradation, where an exothermic peak appeared in the DSC curve. The time interval between the switch from nitrogen to air and the onset of the thermo-oxidation exotherm was reported as the OIT time [26].

#### 2.2.5. Barrier Properties

Representative PCR-PP EBM bottles were selected as being free of defects, serving as representative samples of each treatment in three repeated measures. The oxygen transmission rate (OTR) values were determined using ASTM D3985-17 [27] using a Mocon Ox-Tran Model 2/21 (AMETEK MOCON Inc., Brooklyn Park, MN, USA). The OTR was measured in cm^3^/pkg-day at atmospheric O_2_ and 23 °C. The water vapor transmission rate (WVTR) was measured as g/pkg–day at 90% RH, 37.8 °C, and 10 standard cubic centimeters per minute (sccm), according to ASTM F1249-20 [28] using a Permatran W Model 3/33 (AMETEK MOCON Inc., MN, USA).

#### 2.2.6. Mechanical Properties

The tensile properties (modulus of elasticity and yield stress) of virgin PP and post-consumer PP EBM bottles were examined using a Shimadzu Autograph AGS-J Tensile Tester (Shimadzu Instruments Manufacturing, Co., Ltd.; Analytical & Measuring Instruments Division, International Operations Department 1-3, Kanda Nishiki-cho, Chiyoda-ku, Tokyo 101-8448, Japan). The method used to determine mechanical properties was ASTM D638-14 (standard test method for tensile properties of plastics) [29]. The test specimens sectioned from bottles as strips, using a calibrated 2.54 cm wide shear, were then attached to the grips with 50 mm separation and separated at a rate of 50 mm/min. Each treatment has five repeated measures.

### 2.3. Suitability of Post-Consumer PP EBM Bottles for Direct Food Contact

#### 2.3.1. Heavy Metal Analysis

Three samples of 0.1500 ± 0.0005 g were collected from pelletized MRF-recovered PCR-PP and virgin PP resins with three repeated measures. Samples were digested using microwave digestion (milestone UltraWave) in 5 mL HNO_3_ (Thermo Scientific 67% *v*/*v* Trace Metal Grade) and 1 mL HCl (Thermo Scientific 34% *v*/*v* Trace Metal Grade). Digested samples were evaluated for the presence of Al (aluminum), Cd (cadmium), Cr (chromium), Fe (iron), Pb (lead), Sb (antinomy), and Ti (titanium) using Inductively Coupled Plasma—Optical Emission Spectrometry (ICP-OES, Thermo Scientific iCap-7400 Duo).

#### 2.3.2. CFR Analysis and Cramer Classification

Di-ethyl phthalate (DEP), di-isobutyl phthalate (DIBP), di-butyl phthalate (DBP), Dipentyl phthalate (DPENP), dihexyl phthalate (DHEXP), di-cyclohexyl phthalate (DCHP), di-(2-ethylhexyl) phthalate (DEHP), Diisononyl phthalate (DINP), diisodecyl phthalate (DIDP), Bisphenol-F (BPF), Bisphenol-A (BPA), Bisphenol-B (BPB), Bisphenol-S (BPS), Bisphenol A diglycidyl ether (BADGE), benzophenone and Ethyl-P-Toluate were purchased from Sigma-Aldrich (St. Louis, MO, USA), with purity higher than 99%. Acetone (Fisher Scientific Inc., Fair Lawn, NJ, USA), xylene (Fisher Scientific, Hanover Park, IL, USA) were HPLC grade. The water used was purified using a Milli-Q gradient A10 system (Billerica, MA, USA).

**Figure 3:**
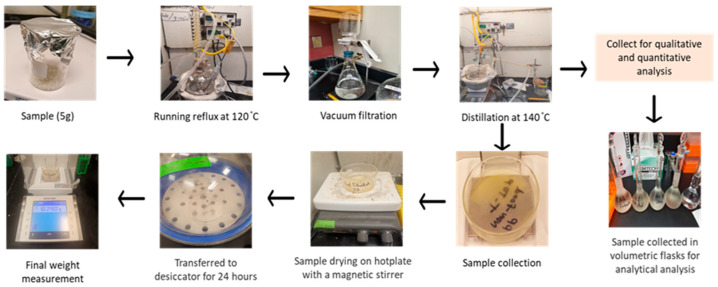
CFR and analytical analysis of polypropylene sample.

All the materials (spatula, scissors, glass materials) in direct contact with the sample were washed with a precision detergent (Alconox, Inc, New York, NY, USA), deionized water and acetone (HPLC-grade), then dried in the oven at 150 °C and covered with aluminum foil until use to avoid cross-contamination. The use of plastic materials for extraction and material handling was strictly avoided. The samples were shredded into small pieces (2–5 mm) using a grinder. To extract the polypropylene samples, the Code of Federal Regulations (CFR) 21 part 177.1520 was followed [30]. Each sample (5 g) was dissolved in xylene (1 L) at 120 °C for 2 hr, then cooled to room temperature. The precipitated polymer was vacuum-filtered at room temperature and the filtrate was distilled at 140 °C until approximately 100 mL remained. The concentrated filtrate was added to a pre-weighted petri dish for extractable measurement. The sample containing petri dish was transferred to a hot plate for residual solvent evaporation and then stored in desiccators for 24 h. The extractable content was calculated based on Equation (1). A similar CFR extraction approach was followed (Figure 3) until the distillation and solvent reduced to 100 mL; then, it was collected for the gas chromatographic analysis. All samples were extracted in triplicate.

(1) $$\%\;w/w=\frac{\mathrm{weight}\;\mathrm{of}\;\mathrm{extractables}}{\mathrm{initial}\;\mathrm{weight}}$$

Both virgin and PCR PP bottle extracts were analyzed by GC-MSD in full scan mode for the unknown identification using gas chromatography and mass spectrometry (HP 6890 series GC system with an auto-sampler and HP Agilent 5973 mass selective detector). The detailed parameters are provided in Table S2 (Supplemental Materials). Compound identification was carried out using a mass spectral library (National Institute of Standards and Technology (NIST) version 2021). A threshold of reverse fit >600 and forward fit >600 from the NIST library was utilized for the identification of unknowns as per NIST recommendations [31]. The Cramer decision tree [32] was used to generate a structure-based Threshold of Toxicological Concern and classify organic chemicals into three classes of hazard level: low (I) toxicity, intermediate (II) toxicity and high (III) toxicity. Cramer class examines the presence of specific functional groups associated with known toxic effects and determines the hazard level [33].

To quantify the presence of the 9 phthalates and 5 bisphenols, stock standards were prepared in a cocktail at 1000 μg/mL. The internal calibration curve was made by serial dilution with dilution factor and followed by addition of the internal standard Ethyl p-Toluate (EPT). This was maintained at a constant 5 μg/g concentration, then analyzed by GC–QqQ- GC-MS/MS; Agilent 7000, Triple Quad, GC 7890A using multiple reaction monitoring (MRM) conditions (Table S2). The standards for the calibration curve and the samples were analyzed with the method mentioned above. BSTFA (N, O bis(trimethylsilyl)trifluoroacetamide) was used as the derivatization agent. A total of 50 μL of BSTFA and 100 μL of acetone (to accelerate the reaction rate) was added to each blank, standard, and sample before analysis. The LOD and LOQ were determined following the [34] internal calibration method. This process was performed using the standard deviation (SD) of the response and the slope of the calibration curve. The LOD was calculated as 3.3 SD/slope (μg/g) and the LOQ was 10 SD/slope (μg/g).

### 2.4. Statistical Analysis

Data were analyzed using one-way ANOVA considering a 95% confidence level (α = 0.05) on JMP^®^ 16 Pro (SAS Institute., Cary, NC, USA) to determine significant differences between treatment means. Pairwise comparison was carried out using the Tukey’s honestly significant difference (HSD) test.

## 3. Results and Discussions

### 3.1. Physical Performance of MRF-Recovered PCR-PP EBM Bottles

#### 3.1.1. Polymer Viscosity

**Figure 4:**
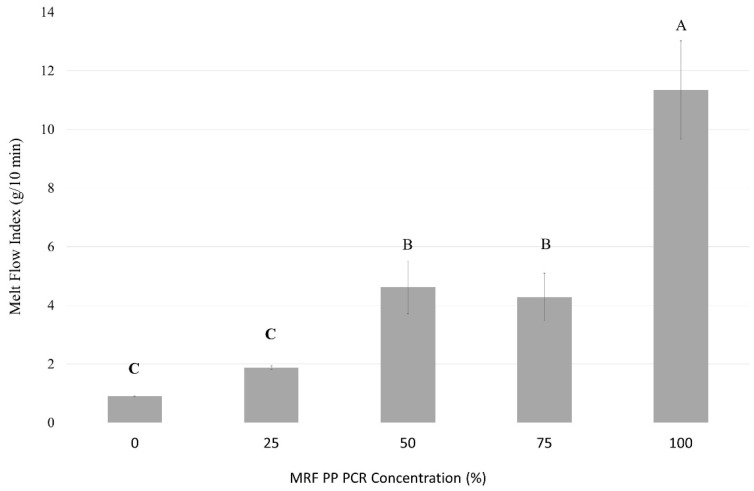
Melt flow rate of PP EBM bottles with varying weight percentages of MRF-recovered PCR-PP. There is evidence of a difference in measured average MFI among treatments (*p*-value < 0.05). Average values (±standard deviation) with the same letters (A, B, C) between MRF PP PCR concentrations are not significantly different (*p*-value < 0.05, Tukey test N = 15).

The melt flow rate is a rapid and convenient measurement technique commonly used during manufacturing for raw material quality control and qualification before processing. MFR is strongly dependent on the polymer molecular weight [35]. The MFR is associated with polymer’s molecular weight, viscosity, and flow of the polymer, providing valuable information on polymer processability and its intended application [36]. Previous research by Curtzwiler et al. determined that the addition of recycled content increased measured MFR, indicating a lower viscosity [22]. This could be attributed to the increases in molecular weight distribution and lower chain molecular weight caused by reprocessing and prior utilization. The data collected herein (Figure 4) suggest that the changes in MFR depend more on the type of virgin and PCR-PP. Bottles with 50% and 75% PCR-PP did not show significant differences between the different ratios of PCR-PP added to the blend; however, they were significantly different than 100% PCR-PP. The virgin PP presented an MFR value almost 12 times lower than 100% PCR-PP. The MFR increased with PCR-PP concentration. Eriksen et al. explained MFR values between 0 and 1 g/10 min are often recommended for extrusion applications, whereas blow-molding requires 0.3 and 5 g/10 min and injection-molding values must be higher than 5 g/10 min. MFR values between 5 and 50 g/10 min can be applied to mold thicker-walled products [37].

#### 3.1.2. Polymer Molecular Structure

**Figure 5:**
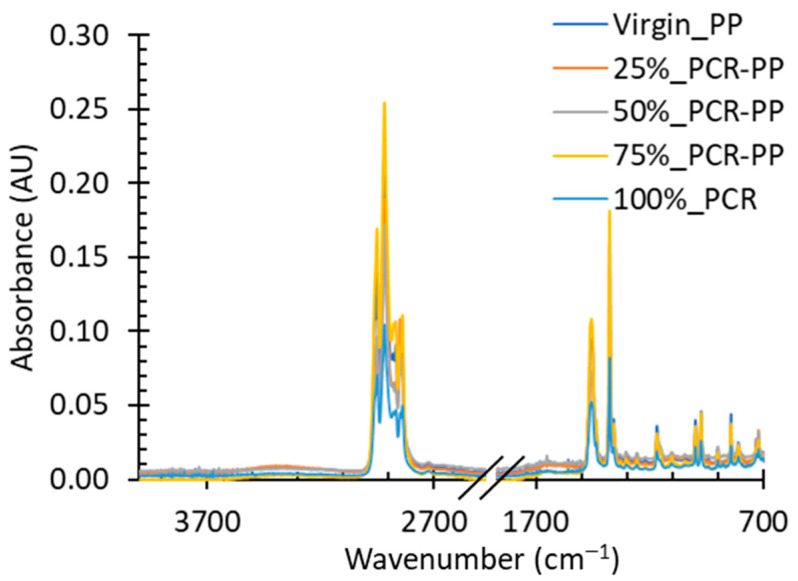
FTIR spectra of selected PP EBM bottles with varying weight percentages of MRF recovered PCR-PP.

FTIR was used to compare virgin PP and PCR-PP blends to understand changes in molecular interactions and molecular composition. Figure 5 shows an overlay of representative examples of the collected spectra. Characteristic bands at 2949 cm^−1^ and 2916 cm^−1^ indicate C-H bond stretching (aliphatic hydrocarbon) [38] and bands at 1450 cm^−1^ and 1375 cm^−1^ are representative of asymmetric and symmetric C-H deformations [39]. All spectra possess bands that are characteristic of PP with no noticeable blue or red shifting in the characteristic bands, indicating similar chemical environments [38].

#### 3.1.3. Thermal Properties

**Figure 6:**
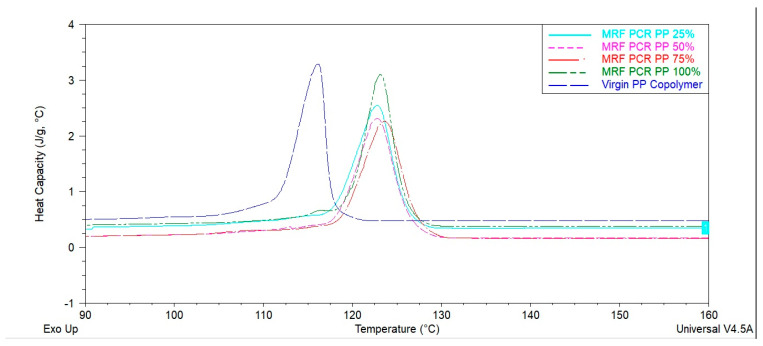
Heat capacity of PP EBM bottles with varying weight percentages of MRF-recovered PCR-PP.

**Figure 7:**
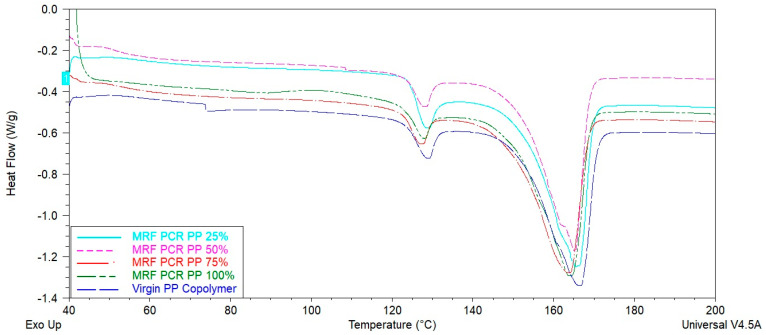
Heat flow of PP EBM bottles with varying weight percentages of MRF-recovered PCR-PP.

Representative heat flow thermograms of all samples are shown in Figure 6 and Figure 7. The Tm (melting temperature), Tc (crystallization temperature), and Xc (crystallinity; %) values of virgin PP and PCR-PP at different percentages are presented in Table 2. The Tc increased with the addition of PCR-PP to the virgin PP; this is beneficial, as higher crystallization temperatures can result in reduced cycle times and increased production rates. This observation can be attributed to residual nucleating agents in the post-consumer material and suggests that fewer additives may be required in PCR blends to achieve the same effect [40,41,42,43,44].

The statistical analysis of the Tm values of the virgin PP and PCR-PP samples did not possess significant differences in comparison to either the first or second heat cycle, indicating that crystal quality was not influenced by the presence of the MRF-recovered PCR-PP. However, the ΔHm1 (melting enthalpy) values increased as the percentage of PCR-PP in the material increases and the 100% PCR-PP was statistically the highest; however, the melting enthalpy of the second heat cycle was statistically the same for all blends. These results suggest faster crystallization rates during the bottle manufacturing process, which are supported by the higher crystallization temperatures of PCR-PP-containing blends. The melting enthalpy values for virgin PP (49.1 J/g for Hm1 and 49.7 J/g for Hm2) are lower compared to the highest PCR-PP percentages. Due to the increase in melting enthalpy values with PCR-PP-loading into virgin PP, the degree of crystallinity of blends gradationally increased in the blends as manufactured.

#### 3.1.4. Thermooxidative Stability of Post-Consumer PP EBM Bottles

**Figure 8:**
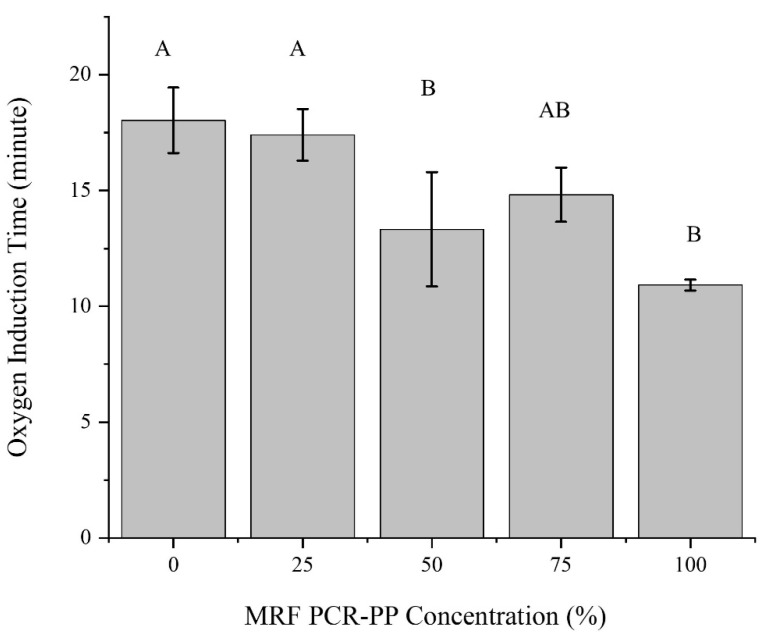
Oxygen induction time of PP EBM bottles with varying weight percentages of MRF-recovered PCR-PP. Note: values with the same letter in a column are statistically the same (α = 0.05).

The oxygen induction time is often used to investigate polyolefin stability, which degrades when heated in an oxidizing atmosphere such as air. The relative antioxidant efficiency in a material is verified by measuring the OIT [35]. The DSC-based OIT thermograms for virgin PP and PCR-PP blends (containing 0, 25, 50, 75 and 100 wt%, respectively) are shown in Figure S1 (Supplementary Materials). The exotherm onset is considered the oxidation initiation [45]. As anticipated, the virgin PP requires the most amount of time for the onset of oxidation to occur, as it would possess the highest concentration of unused antioxidant since it was not exposed to the environment in its first service life and was only subjected to one melt processing cycle. There appears to be sufficient antioxidant in the virgin material and enough remaining in the PCR-PP in the 25% PCR-PP blend, as the OIT of this blend was statistically the same as virgin PP (Figure 8) [26]. These results, coupled with the increased crystallization temperature, suggest that residual additives in post-consumer plastics, coupled with those in virgin plastic, can enable a similar performance to virgin plastics with a lower cost due to the synergistic effect of combining additives. The OIT results are also anticipated to translate into the mechanical properties, as noted by Martin and De Paoli, where the mechanical property degradation from melt-processing cycles was reduced for the stabilized formulations [35].

#### 3.1.5. Barrier Properties

**Figure 9:**
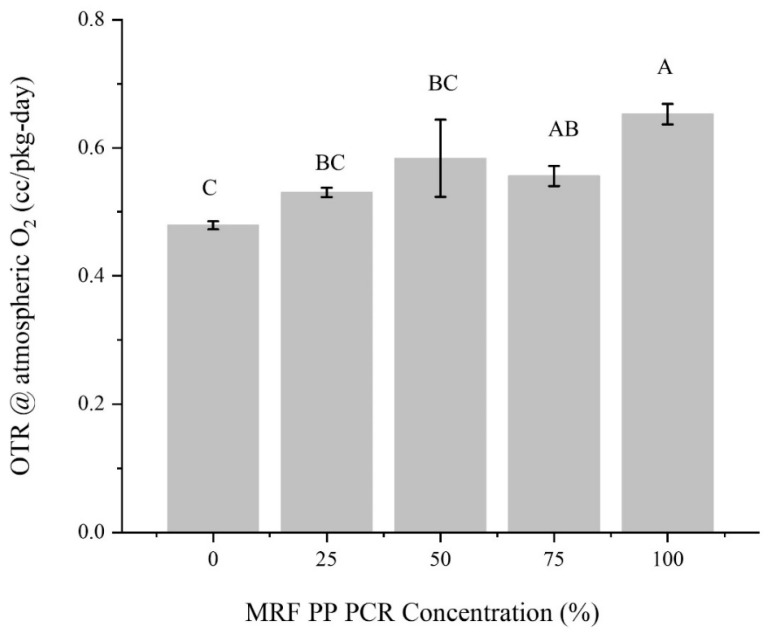
Oxygen transmission rates of PP EBM bottles with varying weight percentages of MRF-recovered PCR-PP. There is evidence of differences in measured average OTR among treatments (*p*-value < 0.05). Average values (±standard deviation) with the same letters (A, B, C) between MRF-recovered PCR-PP concentrations are not significantly different (*p*-value < 0.05, Tukey test N = 15).

A gas barrier is defined as the resistance of packaging to the diffusion and sorption of a substance, which can be expressed in terms of permeability [46]. Two of the most important barrier properties in food packaging are the OTR and WVTR [19]. Oxygen permeation through the packaging results in an oxidation process that causes food spoilage. Thus, reducing the packaging material’s oxygen permeation rate can contribute to increasing product shelf life and maintaining food quality. Virgin PP bottles had the lowest average OTR value of 0.48 ± 0.01 cc/pkg-day, and were significantly different from those with a 75 and 100% concentration of MRF-recovered PCR-PP (Figure 9). From published studies, crystallinity has a significant effect on the gas-barrier properties of polymer materials [47,48]. To improve polymer barrier properties, crystallinity must be higher; however, our materials possessed decreased barrier properties with slight increases in crystallinity due to the increased PCR-PP concentrations. This observation suggests that additional interactions in the polymer’s amorphous fraction contributed to the more facile gas migration through the polymer. Among all treatments, bottles with 100% PCR-PP had the highest average OTR value of 0.65 ± 0.02 cc/pkg-day, corresponding to a 36% increase and significantly different from virgin PP. Also, bottles with 25 and 50% PCR-PP concentrations were comparable and not statistically different from virgin PP bottles’ oxygen transmission rates, with values ranging from 0.48 to 0.58 cc/pkg–day. Thus, it is hypothesized that the decrease in oxygen barrier property mainly relates to pigments, fillers, and contaminants, as observed by the measured concentration of Al, Fe and Ti results from MRF-recovered PCR-PP (Table 3) which are known to significantly affect the crystallinity and performance properties of polymers.

**Figure 10:**
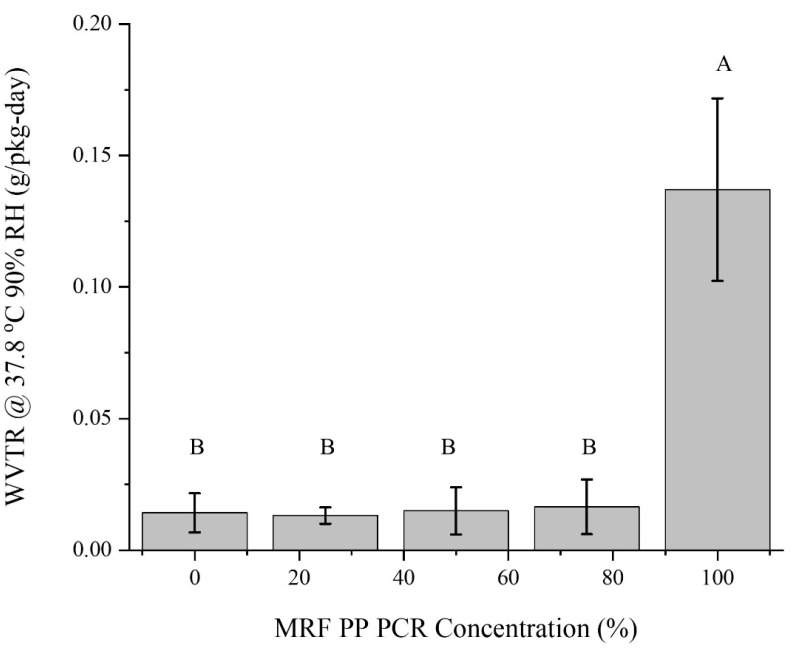
Water vapor transmission rates of PP EBM bottles with varying weight percentages of MRF-recovered PCR-PP. There is evidence of differences in measured average WVTR among treatments (*p*-value < 0.05). Average values (±standard deviation) with same letters (A, B, C) between MRF-recovered PCR-PP concentrations are not significantly different (*p*-value < 0.05, Tukey test N = 15).

The WVTR indicates how easily moisture vapor can permeate a packaging structure. When maintaining a safe moisture content for products during the storage period, a bottle should have a sufficiently low WVTR value. PP is known for its good-to-excellent water vapor barrier properties, as reflected in the results of this study. Mixing virgin PP resin with 25–75% concentrations of MRF-recovered PCR-PP did not significantly change the water-vapor barrier properties of virgin PP. Figure 10 shows the measured average WVTR for the five PCR-PP concentrations. The 100% MRF-recovered PCR-PP bottle had the highest value of 0.14 ± 0.03 g/pkg–day and was significantly different from all other treatments. On the other hand, bottles with 100% virgin PP and 25–75% post-consumer PP did not show significant differences, with an average WVTR ranging from 0.01 to 0.02 g/pkg-day.

The results of this study indicate that adding PCR-PP to virgin PP in EBM bottles decreases its ability to limit oxygen and water-vapor transmission, as reflected by the increasing trend of the transmission rates. However, these increases were only from 5% to 36% different, suggesting that PCR-PP can be incorporated into products without detrimental impacts on barrier performance. A previous study [19] reported the opposite trend regarding the effect of mixing PCR polyolefins in virgin PP resin, where it was reported that 100% post-consumer recycled polyolefin resulted in increased barrier properties (both for oxygen and water vapor) compared to virgin polypropylene. However, it is important to note that the barrier properties of a material can be affected by its chemical structure and processing parameters during production and the material’s grade, quality, purity, filler composition, etc. [48].

#### 3.1.6. Tensile Properties

Mechanical properties are a measure of materials’ behavior when subjected to stress or deformations. Plastic bottles, when used, must have sufficient strength to maintain physical integrity during the handling, transport, and storage of products. These properties include the modulus of elasticity (MOE), flexibility, hardness, and yield stress. MOE is the property that describes the stiffness of the polymer, while yield stress is the point where strain increases and no change in stress and plastic deformation occurs.

**Figure 11:**
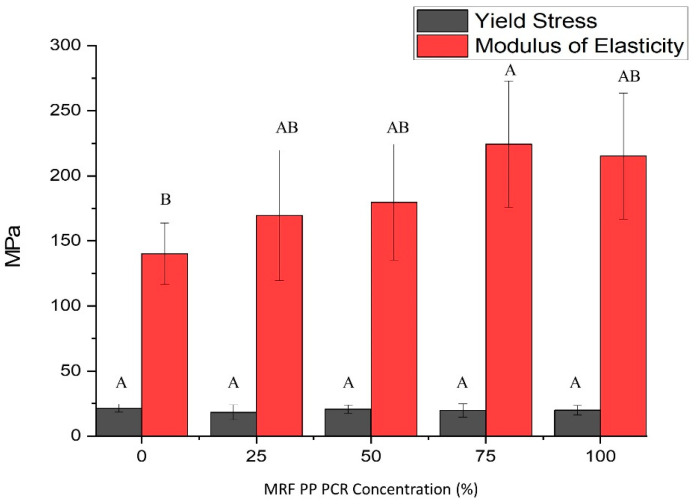
Tensile properties of PP EBM bottles with varying weight percentages of MRF-recovered PCR-PP. There is evidence of differences in measured average modulus of elasticity among treatments (*p*-value < 0.05). Average values (±standard deviation) with same letters (A, B) between MRF-recovered PCR-PP concentrations are not significantly different (*p*-value < 0.05, Tukey test N = 25).

Figure 11 shows that the MOE of PP EBM bottles differs significantly among the different compositions of PCR-PP (*p*-value < 0.05). Bottles with 75% PCR-PP had the highest MOE value of 224 ± 49 MPa, which was significantly higher than virgin PP bottles with an MOE of 140 ± 24 MPa. Variability in measured MOE could be caused by differences in the presence of impurities. Measured average yield stress ranging from 18 to 21 MPa did not show significant differences among treatments. These results indicate that introducing PCR-PP increased the stiffness, but the yield stress remained the same, providing better performance during transport and shipping.

### 3.2. Compliance of MRF-Recovered Post-Consumer PP EBM Bottles

#### 3.2.1. Metals’ ICP-OES

MRF-recovered PCR-PP EBM bottle samples were analyzed for common metals such as Al, Sb, Cd, Cr, Fe, Pb and Ti because these are used as catalysts during the polymerization process. Also, according to the Coalition of Northeastern Governors (CONEG), the sum of Cd, Cr^+6^, Hg, and Pb should not exceed 100 ppm [17]. From the results, Cd, total Cr, and Pb were not detected in the MRF-recovered PCR-PP but were detected in virgin PP resin with a sum < 100 ppm. Mercury was not detected in the MRF-recovered PP using X-ray fluorescence spectroscopy (LOD = 1 ppm). These results suggest that the PP EBM bottles with post-consumer PP would comply with current CONEG metals legislation. Table 3 shows the metals measured for virgin PP resin and pelletized MRF-recovered PCR-PP. It was observed that Al, Fe, and Ti were the main materials present in both samples, which may be attributed to the additives, fillers, catalysts, or contaminants in the samples. No Sb, Cd, Cr, or Pb were detected in post-consumer PP.

#### 3.2.2. 21 CFR 177.1520 Extraction for Extractable and Cramer Classification

**Figure 12:**
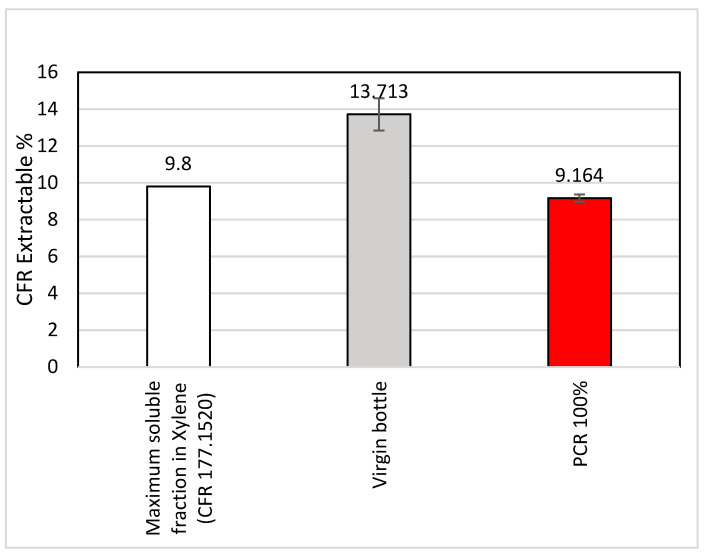
Extractable % virgin PP and MRF-recovered post-consumer PP samples.

The virgin and MRF-recovered PCR-PP were characterized for compliance with the Code of Federal Regulations for direct food contact via 21 CFR 177.1520. According to CFR 21, part 177.1520, the extractable fraction from polypropylene should not exceed 9.8% in weight [30]. The virgin PP comprised a higher percentage of extractable matter (13.713%) compared to 100% PCR-PP (9.164%); this was anticipated, as the virgin PP was not food-grade (Figure 12). In our experiment, the virgin PP exceeded that specification limit, while the PCR-PP was within the specification limit. In general, virgin PP has a higher molecular weight and crystallinity than the same PCR-PP polymer after its first service life and the recycling process due to the multiple heating treatments that occur during the recycling process, where a wide range of thermal and oxidative degradation occurs [48,49]. The extractables from a polymer can vary depending on various factors, for example additives, purity, oligomeric content, and the processing method (extrusion, recycling, molding, etc.) [50]. Another possibility is the alteration or degradation of the additives or fillers in the PCR sample during the recycling process, which is absent in the virgin sample, and can reduce the extractable content in the PCR-PP sample. While the virgin PP was non-food-grade PP, the MRF-recovered PP was likely composed of a mixture of food-grade and non-food-grade, which could explain the lower CFR extractables for the MRF-recovered material [30].

The results for non-intentionally added substances (NIAS) and related Cramer classification-based toxicity levels are listed in Table 4. NIAS are substances that may be present in packaging polymers with no known application and may pose a risk to health if they migrate into food [51]. Analysis indicated that PCR samples contained more additives, pigments, and impurities than virgin PP. Substances only detected in the PCR sample, such as myristic acid, oleic acid [52], stearic [53], 1-monopalmitin, have applications as lubricants, emulsifiers, or surfactants in the industry. Glycerol monostearate is used as an antistatic additive in polypropylene and in the food sector as an emulsifier [54]. Myristic acid has diversified applications as a flow agent and emulsifier in the food sectors, as a surfactant in soaps, detergents, and as an internal or external lubricant in plastics (ACME-Hardesty). The substances found only in PCR samples may have been sourced as additives to improve flexibility and compatibility, or as contaminants from other packaging or waste during the first intended service life. Butylated hydroxytoluene (BHT) was only found in the virgin sample, which is generally used as an antioxidant in plastic packaging materials such as polyethylene and polypropylene films [55]. This prevents degradation during the service life. BHT is listed under the Registration, Evaluation, Authorisation, and Restriction of Chemicals (REACH) regulation [56], which is designed to increase safety and improve the protection of human health and the environment from hazardous chemicals.

The compounds detected (8 in virgin PP and 15 in PCR-PP) in both types of samples can often be considered intentionally added substances. For example, m-Toluic acid is used as a dye or polymer stabilizer [57] and palmitic acid, a fatty acid found in both virgin and PCR samples, has applications as a stabilizer in polymers [58]. The Cramer decision tree [32,33] classifies organic chemicals into three classes of hazard level: low (I) toxicity, intermediate (II) toxicity and high (III) toxicity. Cramer class examines the presence of specific functional groups associated with known toxic effects and determines the hazard level [58]. Of the compounds found in the virgin sample, Cramer class I consists of 50%, class II consists of 12.5%, and class III consists of 37.5%. In the PCR sample, 15 chemicals were detected: class I consists of 46.67%, class II consists of 0%, class III consists of 40.0%, and 13.3% was unclassified. Based on the Cramer classification and number of detected compounds, it appears that the PCR sample has a higher probability of toxicity than the virgin sample. However, it is difficult to accurately determine the overall risk severity in the PCR or virgin samples without more information about the specific compounds’ total exposure measurements (concentration and diffusion coefficients) and potential toxicological effects. Recycled plastics, including recycled polypropylene, originating from mixed plastic waste streams often exhibit a wider range of contaminants than virgin materials, specifically recycled PP sourced from non-food-grade materials mixed with food and non-food-grade plastics, which may not comply with stringent regulations and can introduce a wide range of unknown contamination [37].

Phthalates are synthetic chemicals widely used in plastic packaging and industrial products to improve the mechanical properties, as well as providing flexibility and softness [59]. Bisphenols have wide applications in the production of polycarbonate plastics, epoxy resins, adhesives, and several additives [60]. However, they are endocrine disruptors/modulators and hazardous to health at very low concentrations (nanogram level) [61]. Analyzing phthalates and bisphenols in non-food-grade recycled PP is important to understand their compliance with phthalate and bisphenol regulations, as well as emerging corporate restrictive substance lists (RSLs). Information regarding the prevalence of endocrine modulators in PCR is also rare in the literature.

**Figure 13:**
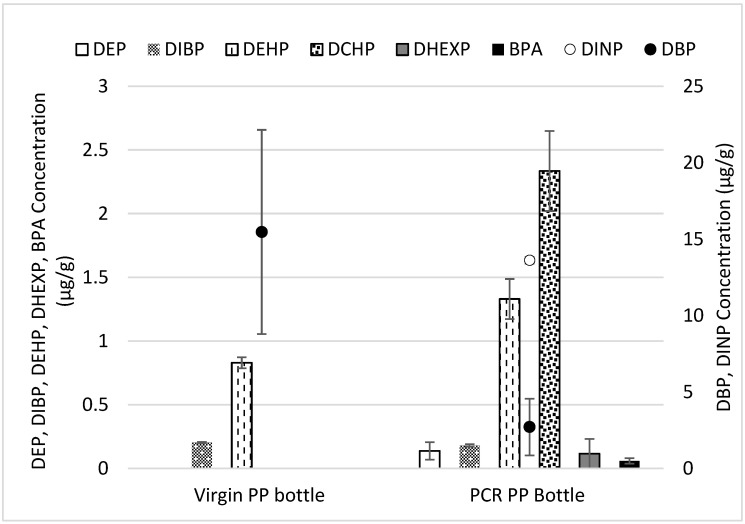
Phthalate and bisphenols in virgin and PCR bottle samples.

In our samples, the virgin PP sample contained diethylhexyl phthalate (DEHP) at a concentration of 0.83 μg/g and in PCR-PP with a concentration of DEHP (1.33 μg/g). While DCHP (2.335 μg/g), DINP (13.617 μg/g), BPA (0.059 μg/g) were only detected in PCR-PP sample (Figure 13). The additional phthalate detected in the PCR samples was as follows: DEP (0.138 μg/g), DIBP (0.181 μg/g), DHEXP (0.116 μg/g), and DBP (2.712 μg/g). In the PCR sample, the concentration of DIBP and DBP (0.181 μg/g and 2.712 μg/g, respectively) was slightly lower compared to the virgin PP sample. Perestrelo et al. investigated various plastic packages from the European market and determined that DIBP was the most prominent phthalate in packaging, with concentrations ranging between 3.61 and 10.7 μg/L [59]. The concentration of DEHP in the PCR sample is higher than virgin at 1.33 μg/g. The higher concentration of DBP in the virgin PP sample suggests its intentional addition during the pellet manufacturing, as no additives were included during bottle manufacturing. However, polymers sourced after recycling can face diversified processing and contamination, which can influence the analyte concentration compared to the virgin sample; this was also prominent in our result. On the other hand, the inherent heterogeneity of recovered post-consumer polymers can contribute to a wide range of standard deviation, which was observed for DBP concentrations. The use of DEHP, DBP, BBP, DIDP, and DINP has a threshold of 0.1% in the final product [62], and Toxics in Packaging Clearinghouse (TPCH) set the limit for incidental ortho-phthalates to no more than 100 μg/g in packaging [63]. Overall, the phthalates detected in the virgin and PCR samples were below the TPCH limit of 100 μg/g and complied with the regulations. Although polypropylene is often considered to be “BPA free” and safe for use as packaging [64], BPA was detected in the PCR sample at a concentration of 0.059 μg/g.

Our findings in this study fill the knowledge gaps related to extractables, NIAS/IAS identification, and targeted extractable compounds from material-recovery-facility-sourced PCR-PP materials. The contamination levels in PCR-PP were higher than the virgin sample, according to Cramer classification and quantification results for certain phthalates and BPA. However, they did not exceed the limits set by the Toxics in Packaging Clearinghouse (TPCH). With increasing worldwide and domestic regulations encouraging and requiring the use of recycled plastic with virgin material, our scientific data can provide valuable information to the scientific community and manufacturers regarding potential uses for MRF-recovered PP. Additionally, the study provides information on common additives, antioxidants, and stabilizers that can be present as non-intentionally added substances (NIAS) in both virgin and recycled PP.

## 4. Conclusions

PP bottles are commonly used to store food products, chemicals, and pharmaceuticals because of their good chemical resistance, superior strength, and cost advantages in blow molding compared to PET [64]. This study investigated the compliance and physical performance of extrusion blow-molded MRF-recovered PCR-PP bottles. Material properties loosely followed the law of mixtures, although some remained statistically the same. This general trend was observed in multiple studies that blended virgin and post-consumer recycled polymers. Therefore, it is important to note that the increase or decrease in properties depends upon both post-consumer and virgin polymers. Key results include:

- Increased crystallization temperature when PCR is present in the blend;
- No practical difference in crystallinity as a function of PCR concentration as-molded (first heat curve);
- No other polymers are present in the thermograms of MRF-recovered PP materials, indicating high polymer purity (melting peaks);
- Oxygen and water-vapor barrier properties remained relatively constant unless the composition was 100% MRF material;
- MRF-recovered PCR-PP significantly (*p*-value < 0.05) improved the stiffness of virgin PP bottles. On the other hand, the measured yield stress for all treatments was significantly similar;
- A wider range of N/IAS was detected in PCR material compared to the virgin material, which can be attributed to plastic additives, food additives, and degradation byproducts;
- Regulatory compliance (limits of TPCH: 100 μg/g) and maintaining properties up to 75% MRF PCR demonstrates the increased value of MRF materials. Targeted phthalates did not exceed the limits of TPCH, and trace levels of BPA were detected in the MRF PCR-PP.

The results of this study provide critical information for stakeholders making decisions to use MRF recovered in food packaging applications. Moreover, this study demonstrates the viability of a significant source of PP and its notable long-term impacts, increasing profits by using PCR materials. This approach will produce environmentally responsible food plastic packaging in compliance with legislation in the circular economy. However, it must be noted that the material recovery facility post-consumer polypropylene used in this work is only representative of a single, sorted post-consumer bale from one material recovery facility. The reproducibility and variance in the post-consumer polypropylene properties from material recovery facilities is progressing, specifically for bale sources from urban, suburban, and rural areas.

Future work should continuously monitor new PCR sources from different MRFs to understand consistency regarding the US. One should determine levels of PFAS and phthalates of PCR-PP and other recycled plastics to address the increasing concerns regarding toxicity. An additional analysis using GC tandem mass spectrometry to reduce the limits of detection/quantification is in progress. Lastly, a study on the efficiency of different decontamination processes would be vital to emphasize the impacts of using MRF PCR plastics and their viability for direct food-contact applications.

## Figures and Tables

**Table 1:** Experimental design.

Treatments	% (wt) MRF-Recovered Post-Consumer PP
1	0 (virgin PP)
2	25
3	50
4	75
5	100

**Table 2:** Average thermal properties of PP EBM bottles with varying weight percentages of MRF-recovered post-consumer PP.

Treatment	Tm1 (°C)	Tm2 (°C)	Tc (°C)	Hc (J/g)	Hm1 (J/g)	Hm2 (J/g)	Crystal 1 (%)	Crystal 2 (%)
1	165.5 ^a^	162.1 ^a^	115.9 ^b^	66.2 ^ab^	49.1 ^ab^	49.7 ^a^	23.7 ^ab^	24 ^a^
2	165.9 ^a^	162.3 ^a^	122.8 ^a^	62.6 ^b^	48 ^b^	51.3 ^a^	23.2 ^b^	24.8 ^a^
3	165.3 ^a^	161.2 ^a^	123.2 ^a^	72.6 ^ab^	51.2 ^ab^	51.4 ^a^	24.7 ^ab^	24.9 ^a^
4	164.0 ^a^	161.6 ^a^	123.4 ^a^	76.2 ^a^	54.1 ^ab^	54.8 ^a^	26.1 ^ab^	26.5 ^a^
5	164.8 ^a^	159.3 ^a^	123.1 ^a^	73.6 ^ab^	54.8 ^a^	54.9 ^a^	26.5 ^a^	26.5 ^a^

Note: values with the same letter in a column are statistically the same (α = 0.05).

**Table 3:** Measured metals compliant with CONEG.

	Element Concentration (µg/g or ppm)						
Sample	Al	Sb	Cd	Cr	Fe	Pb	Ti
PP virgin	3.45	* b	0.13	0.17	* b	4.10	* b
PP MRF	84.2	* b	* b	* b	49.02	* b	68.26
MLOD (ppm)	0.124	0.011	0.002	0.003	0.010	0.689	0.002
MLOQ (ppm)	0.41	0.04	0.01	0.01	0.03	2.30	0.01

* b—below limit of detection.

**Table 4:** IAS/NIAS compounds in virgin and 100% PCR-PP bottle sample.

Virgin Sample		100% PCR Sample	
Compound (CAS)	Cramer Class	Compound (CAS)	Cramer Class
M-Toluic acid, TMS derivative (959296-29-8)	Class I	m-Toluic acid, TMS derivative	Class I
Butylated Hydroxytoluene (BHT) (128-37-0)	Class II	1-iodo- Decane (2050-77-3)	Class III
4-cyano-3-fluorophenyl 4-(4-butylcyclohexyl)benzoate (92118-83-7)	Class III	1,1′-(1,2-dimethyl-1,2-ethanediyl)bis- Benzene (4613-11-0)	-
1,1′-(1,2-dimethyl-1,2-ethanediyl)bis Benzene (5789-35-5)	Class III	1′-(1,2-ethanediyl)bis[4-methyl- Benzene	Class III
1′-(1,2-ethanediyl)bis 4-methyl- Benzene (538-39-6)	Class III	Myristic acid, TBDMS derivative (104255-79-0)	Class I
4-Allyl-2-methoxyphenyl benzoate	Class I	Tricyclo[4.4.0.0(2,7)]decan-3-one, 1-methoxy-2,6-dimethyl- (62648-63-9)	Class III
Palmitic Acid, TMS derivative (55520-89-3)	Class I	1,2-Bis(3,5-dimethylphenyl)-diazene 1-oxide (64857-67-6)	Class III
Stearic acid, TMS derivative (18748-91-9)	Class I	Palmitic Acid, TMS derivative (55520-89-3)	Class I
-	-	Linoelaidic acid, tert.-butyldimerthylsilyl ester	-
-	-	Oleic Acid, (Z)-, TMS derivative (21556-26-3)	Class I
-	-	Stearic acid, TMS derivative (18748-91-9)	Class I
-	-	1-Monopalmitin, 2TMS derivative (1188-74-5)	Class III
-	-	2-Monostearin, 2TMS derivative (53336-13-3)	Class III
-	-	4-tert-Octylphenol, TMS derivative (78721-87-6)	Class I
-	-	Glycerol monostearate (GMS), 2TMS derivative (1188-75-6)	Class I

## Data Availability

Data are contained within the article or Supplementary Material.

## Supplementary Materials

The following supporting information can be downloaded at: https://www.mdpi.com/article/10.3390/polym15163471/s1, Figure S1: DSC OIT thermograms of (a) virgin PP copolymer (b) MRF PCR-PP 25% (c) MRF PCR-PP 50% (d) MRF PCR-PP 75% (e) MRF PCR-PP 100%; Table S1: optimized EBM parameters for post-consumer PP bottle processing; Table S2: parameters of gas chromatographic analysis.
