# Supplementary material for: Suitability of MRF Recovered Post-Consumer Polypropylene Applications in Extrusion Blow Molded Bottle Food Packaging
Source: Polymers (Basel). 2023 Aug 19;15(16):3471. doi: 10.3390/polym15163471 (PMC10458224; doi:10.3390/polym15163471)
Supplement: Supplementary file 1 [file polymers-15-03471-s001.zip › polymers-2551160-supplementary.pdf]

polymers

Supplementary Materials

List of Abbreviations

ANOVA (Analysis of Variance)

ASTM (American Society for Testing and Materials)

ATR-FTIR (Attenuated Total Reflectance Fourier-Transform Infrared Spectroscopy)

BHT (Butylated Hydroytoluene)

BPF (Bisphenol-F)

BPA (Bisphenol-A)

BPB (Bisphenol-B)

BPS (Bisphenol-S)

BADGE (Bisphenol A diglycidyl ether)

CAGR (Compound Annual Growth Rate)

CFR (Code of Federal Regulations)

CONEG (Coalition of Northeastern Governors)

CRD (Complete Randomized Design)

DEP (Di-ethyl phthalate)

DIBP (di-isobutyl phthalate)

DPENP (di-butyl phthalate (DBP), Dipentyl phthalate)

DHEXP (dihexyl phthalate)

DCHP (di-cyclohexyl phthalate)

DEHP (di-(2-ethylhexyl) phthalate)

DINP (Diisononyl phthalate)

DIDP (diisodecyl phthalate)

DSC (Differential Scanning Calorimetry)

EBM (Extrusion Blow Molding)

EPA (Environmental Protection Agency)

EPR (Extended Producer Responsibility)

EPT (Ethyl P-Toluate)

Hm (Melting Enthalpy)

HSD (Honestly Significance Difference)

ICP-OES (Inductively Coupled Plasma-Optical Emission Spectrometry)

LDPE (Low Density Polyethylene)

LOD (Limit of Detection)

LOQ (Limit of Quantification)

MOE (Modulus of Elasticity)

MRM (Multiple Reaction Monitor)

MRF (Material Recovery Facility)

NaOH (Sodium Hydroxide)

NIST (Natiuonal Institute of Standards and Technology)

N/IAS: Non-Intentionally/ Intentionally Added Substances. Definition: Substances that are present in a product or material as a result of the manufacturing process, recycling but are not deliberately added for a specific function (Non-

intentionally added). Substances that are present in a product or material and are deliberately added for a specific function (intentionally added).

OIT (Oxygen Induction Time)

OTR (Oxygen Transmission Rate)

PCR (Post-Consumer Recycled)

PE (Polyethylene)

PET (Polyethylene terephthalate)

PFAS (Per- and polyfluoroalkyl substances)

PP (Polypropylene)

PS (Polystyrene)

PVC (Polyvinyl Chloride)

REACH (Registration, Evaluation, Authorisation, and Restriction of Chemicals)

SD (Standard Deviation)

Tm (Melting Temperature)

TPCH: Toxics in Packaging Clearinghouse. Definition: is an organization that promotes the implementation of state laws that prohibit the use of heavy metals such as lead, mercury, cadmium, hexavalent chromium, phthalates, PFAS etc. in product packaging materials.

WVTR (Water Vapor Transmission Rate)

Table S1. Optimized EBM parameters for post-consumer PP bottle processing

Temperature zone setting	°F	Other parameters	Seed
Rear	365	Screw speed (RPM)	75
Transition	370	Clamp 1 close	80
Metering	370	Clamp 2 close	15
Head 1	375	Clamp open	70
Head 2	375	Clamp daylight	30
Molds	~68	Clamp delay	10
Air pressure (psi)	90	Shuttle left	120
Pre-blow (psi)	10	Shuttle right	120
Cutter heat (%)	≤45	Screw ideal	13

Table S2: Parameters of gas chromatographic analysis

Parameter	Value
**GC-MSD** Carrier gas:	He
Column:	Agilent DB-5ms (250 µm x 0.25 µm film thickness)
Oven Temperature (start):	40 °C (5 min)
First Ramp:	7 °C/min until 220 °C (hold 0 min)
Second Ramp:	7 °C/min until 300 °C (hold 3 min)

Oven Temperature (end):	350 °C
Injection Volume:	1µl
MSD Transfer Line:	300°C
Scan parameter, mass-to-charge ratios (m/z):	40-500
Acquisition Mode:	Full scan, Splitless
**GC-QqQ**	
He Quench Gas:	2.25 mL/min
N2 Collision Gas:	1.5 mL/min
Column:	Agilent DB-5MS, 30 m x 250 µm x 0.25 µm
Oven Temperature (start):	40°C
First Ramp:	30 °C/min until 120 °C (hold 1 min)
Second Ramp:	7.5 °C/min until 300 °C (hold 5 min)
Oven Temperature (end):	300°C
Injection Volume:	1µl
MSD Transfer Line:	300°C
Acquisition Mode:	Multiple reaction monitoring (MRM)

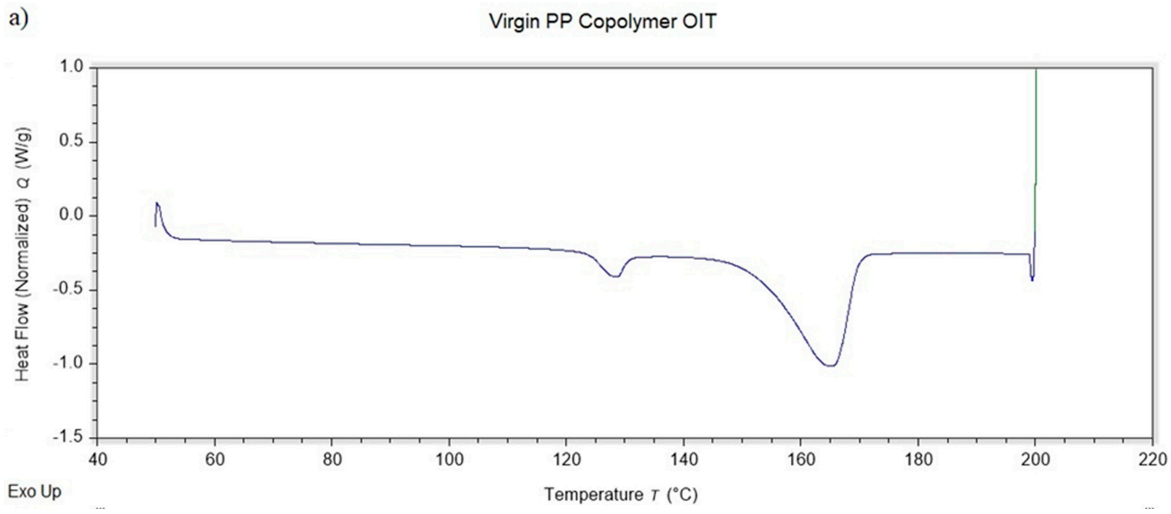

b)

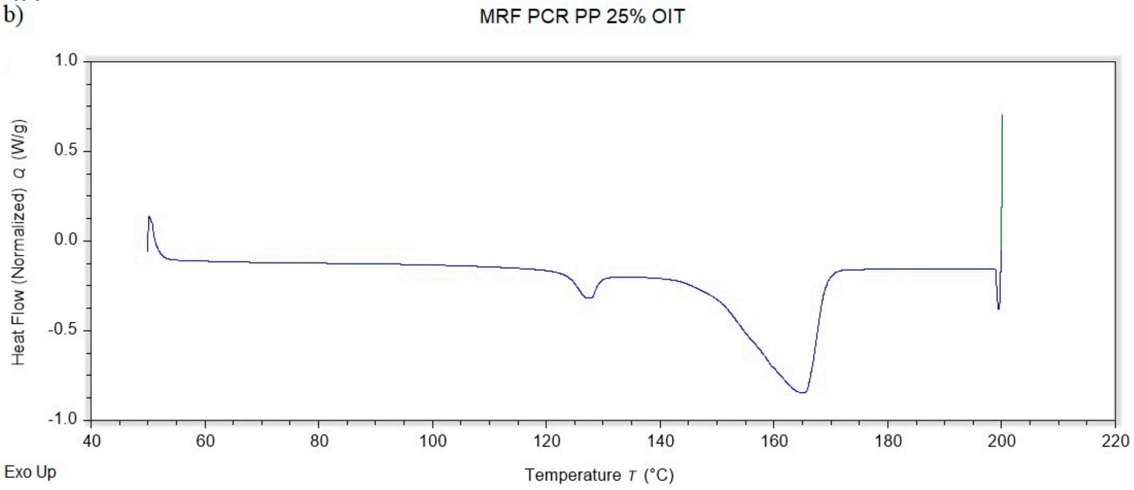
MRF PCR PP 25% OIT
Heat Flow (Normalized) Q (W/g)
1.0
0.5
0.0
-0.5
-1.0
40
60
80
100
120
140
160
180
200
220
Exo Up
Temperature T (°C)

c)

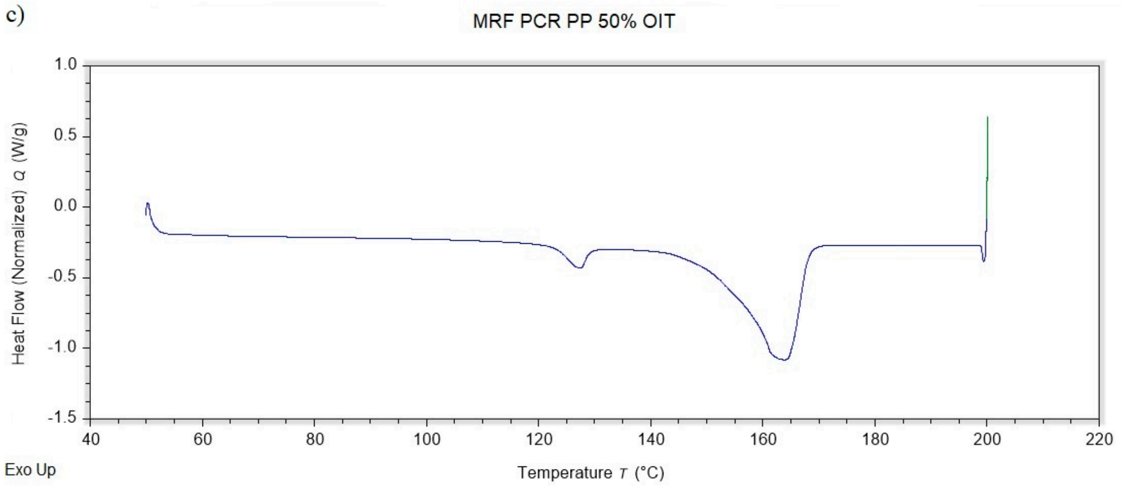
MRF PCR PP 50% OIT
Heat Flow (Normalized) Q (W/g)
1.0
0.5
0.0
-0.5
-1.0
-1.5
40
60
80
100
120
140
160
180
200
220
Exo Up
Temperature T (°C)

d)

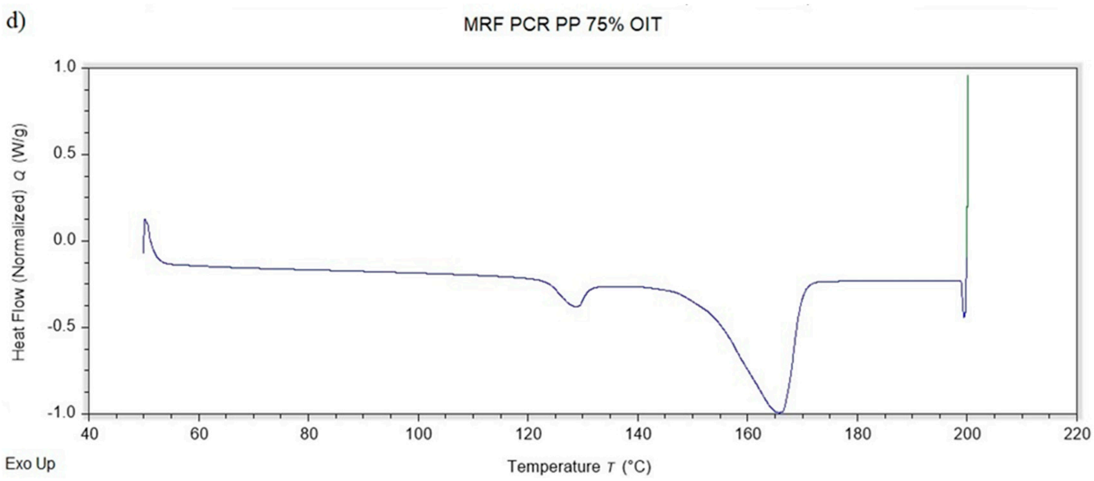

e)

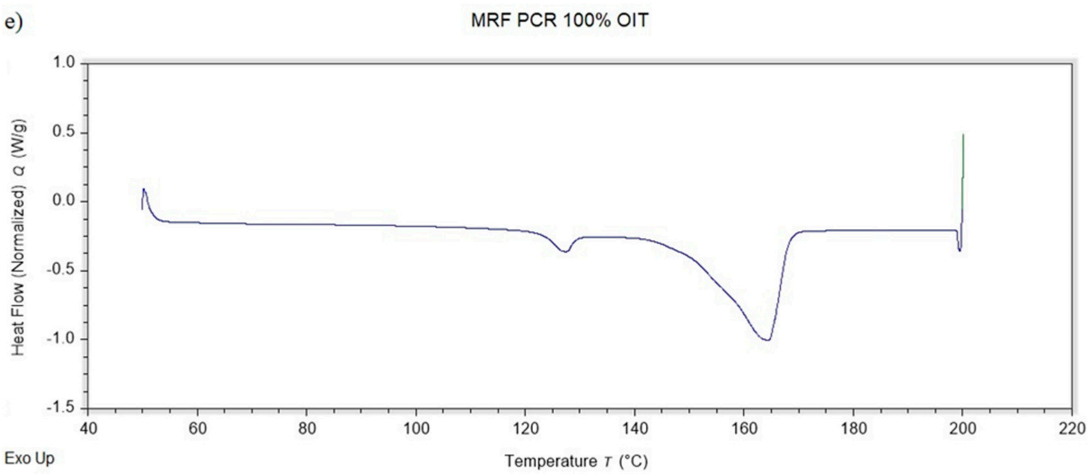

Figure S1: DSC OIT thermograms of a) Virgin PP Copolymer b) MRF PCR-PP 25% c) MRF PCR-PP 50% d) MRF PCR-PP 75% e) MRF PCR-PP 100%
